# Use of immune checkpoint inhibitors in solid organ transplant recipients with advanced cutaneous malignancies

**DOI:** 10.3389/frtra.2023.1284740

**Published:** 2023-10-30

**Authors:** Stephanie Ji, Hao Liu, Laura Pachella, Ryan D. Stephenson, Roman Groisberg, Sarah A. Weiss

**Affiliations:** ^1^ Department of Medicine, Rutgers Robert Wood Johnson Medical School, New Brunswick, NJ, United States; ^2^ Department of Biostatistics and Epidemiology, Rutgers School of Public Health, New Brunswick, NJ, United States; ^3^ Division of Medical Oncology, Rutgers Cancer Institute of New Jersey, New Brunswick, NJ, United States

**Keywords:** immune checkpoint inhibitor, solid organ transplant, melanoma, cutaneous squamous cell carcinoma, merkel cell carcinoma

## Abstract

**Background:**

Immune checkpoint inhibitors (ICI) are standard of care therapy for patients with cutaneous malignancies, the most frequently diagnosed cancers in solid organ transplant (SOT) recipients. The activity and rate of allograft rejection in SOT recipients with advanced skin cancers treated with ICI is understudied.

**Methods:**

We conducted a retrospective analysis of SOT recipients with advanced melanoma, cutaneous squamous cell carcinoma (cSCC), and merkel cell carcinoma (MCC) who were treated with ICI. Unpublished cases from our institution and published cases from the literature were aggregated. Demographics, type of immunosuppressive therapy, type of ICI(s) administered, prior systemic therapies, tumor response to ICI, and evidence of organ rejection and/or failure were recorded. Objective response rates (ORR) and rates of graft rejection and failure are reported.

**Results:**

Ninety patients were identified; four patients from our institution and 86 unique patients from a literature review. ORR to first-line ICI for the entire cohort was 41.1% (37/90). ORR by tumor type was 31% (18/58), 64.3% (18/28), and 25.0% (1/4) for melanoma, cSCC, and MCC, respectively. The rate of graft rejection was 37.8% (34/90) with 61.8% (21/34) of these cases progressing to graft failure. Number of immunosuppressive agents (0, 1, 2, or 3) was inversely associated with rate of graft failure.

**Conclusions:**

In this retrospective analysis, ICIs demonstrate clinical activity in SOT recipients with cutaneous malignancies; however, the rate of graft rejection is high. Treatment plans should be individualized through thorough interdisciplinary discussion. Immunosuppressive modifications may be considered prior to starting treatment, but when feasible, enrollment on clinical trials is preferred.

## Introduction

Cancer is one of the leading causes of death in patients who are solid organ transplant (SOT) recipients. In 2022, over 42,000 solid organ transplants were performed in the United States and the total number of transplants recorded surpassed 1 million (1). Over 100,000 patients are now on the transplant waiting list each year (2). SOT recipients have a 2- to 4- fold increased risk of developing cancer compared to the general population (3, 4). This risk is partially attributed to lifelong immunosuppressive drugs that are required to prevent graft rejection as well as infection with oncogenic viruses (5, 6). Skin cancers are the most frequent malignancy among SOT recipients, accounting for almost 40% of post-transplant malignancies (7, 8). When compared to the general population and depending partially on geography, SOT recipients have a 65- to 250- fold increased risk of developing cutaneous squamous cell carcinoma (cSCC), a 2- to 8- fold increased risk of developing melanoma and a threefold increased risk of developing Merkel cell carcinoma (MCC) (6, 9). Identifiable risk factors for development of post-transplant skin cancers may include a history of pre-transplant skin cancer, male sex, white race, ultraviolet radiation exposure, type and dosing of immunosuppression, age greater than 50 at time of transplant, and recipients of thoracic transplants (8, 10).

Immune checkpoint inhibitors (ICIs) have revolutionized cancer therapy, particularly in the management of advanced cutaneous malignancies. Monoclonal antibodies against cytotoxic T-lymphocyte antigen 4 (CTLA-4), programmed cell death 1 (PD-1), and programmed cell death ligand-1 (PD-L1) are approved by the Food and Drug Administration (FDA) for treatment of cSCC, melanoma, and/or MCC (11). ICIs function by blocking the negative regulation of effector T-lymphocytes, allowing for potentiation of anti-tumor immune responses (12). ICIs are standard of care for the management of advanced skin cancers, however the same co-inhibitory signals that ICIs suppress are implicated in maintaining organ tolerance, which has been demonstrated in several pre-clinical studies (13–19). As a result, ICIs have historically been avoided in patients with a history of SOT due to concerns of causing severe, life-threatening graft rejection (20). Furthermore, the impact of immunosuppressive therapies on the efficacy of concurrent ICI therapy is understudied (21). SOT recipients are typically excluded from clinical trials using ICIs, resulting in limited knowledge of their safety and efficacy in this patient population.

While there have been multiple case reports and several retrospective reviews investigating the use of ICIs in SOT recipients (22–69), results are variable regarding antitumor efficacy and rates of graft rejection (70). No reports to our knowledge have solely focused on the experience of SOT patients with advanced skin cancers treated with ICI. The aim of this study is to document the clinical outcomes of SOT patients with advanced skin cancer treated with ICI at our institution and to aggregate it with the available retrospective literature on this population, which we comprehensively compile here. We also aim to identify clinical factors that may be associated with increased risk of allograft rejection.

## Materials and methods

### Single institution retrospective case series

We searched the Rutgers Cancer Institute of New Jersey (CINJ) electronic medical record (EMR) for SOT recipients who developed an advanced cutaneous malignancy and were treated with ICI by the CINJ Cutaneous Oncology group between January 2019 and May 2022. This retrospective study was approved by the Rutgers Institutional Review Board (#2022000836). Information collected on each patient included: demographics including age and sex, type of SOT, type of immunosuppressive therapy for SOT, type of cutaneous malignancy, type of ICI(s) administered, prior systemic therapies, interval time from transplant to ICI administration, tumor response to ICI, and evidence of organ rejection and/or failure.

### Literature review

A systematic search of PubMed and Google Scholar was manually conducted to identify relevant publications up until April 2022, without any date restrictions. Search terms included “checkpoint inhibitor”, “transplant”, “PD-1 inhibitor”, “CTLA-4 inhibitor”, “nivolumab”, “pembrolizumab”, “ipilimumab”, and “cemiplimab”. All types of publications were reviewed, including case reports and case series if they were written in English and reported on the use of ICI in SOT recipients who developed advanced skin cancer. To be included in this study, the articles were required to provide patient-specific information regarding age, sex, tumor type, type of immunosuppression, allograft status after ICI treatment, and the cancer response to ICI treatment. Article titles and abstracts were evaluated for relevance. Eligible articles were reviewed in their entirety to ensure that all inclusion criteria were met. The same data collected from patients in the CINJ EMR were also extracted from the existing publications.

Graft response to ICI treatment was considered to either be “acute rejection,” “graft failure”, or “no rejection”. For the patients who experienced acute rejection, if the allograft was not rescued and no longer functioned according to the author of the paper (i.e., the patient subsequently became reliant on dialysis or experienced liver failure), then the patient was deemed to have graft failure.

Cancer response to first-line ICI was separated into 4 categories: progressive disease (PD), stable disease (SD), partial response (PR), and complete response (CR). Objective response rates (ORR) were calculated as the proportion of patients who had a CR or PR. While the majority of papers explicitly stated the tumor response, there were four patients in which tumor response was determined by the authors based on the paper's descriptions of the patient and one patient in which the tumor response was non-evaluable due to death from graft rejection. Cancer response was determined based on response achieved with first-line ICI.

Data from the published cases were compiled with our previously unpublished single institution retrospective case series of SOT skin cancer patients treated with ICI. Data were stratified based on tumor type, number of immunosuppressive drugs, and tumor response to ICI. Descriptive statistics were used to report tumor response to ICI and rates of graft rejection or failure. Fisher's exact test was used to compare ORR between tumor types, transplant types, rejection rates between number of immunosuppressants and type of ICI, and rejection rates depending on if there was a change in immunosuppression time of ICI initiation.

## Results

### Baseline patient characteristics

Four patients who met inclusion criteria were identified in the CINJ EMR. The literature search yielded 973 articles. Forty-five of the publications described 86 unique patients who met all inclusion criteria. Thus, 90 total SOT patients treated with ICI for advanced skin cancer were included in our final analysis (Supplementary Table S1). Patient characteristics are shown in Table 1. Median age at time of skin cancer treatment with an ICI was 67 years and there were more men (*n* = 68, 75.6%) than women (*n* = 22, 24.4%). Of these 90 cases, most had melanoma (*n* = 58, 64.4%) followed by cSCC (*n* = 28, 31.1%) and MCC (*n* = 4, 4.44%). The most common SOT was kidney (*n* = 67, 74.4%), followed by liver (*n* = 14, 15.6%), heart (*n* = 7, 7.8%), and lung (*n* = 1, 1.1%). One patient had a heart and kidney transplant (1.1%).

**Table 1:** Patient characteristics.

Demographics	Melanoma		cSCC		Merkel cell		All	
	*n*	%	*n*	%	*n*	%	*n*	%
Total	58		28		4		90	
Age (median)	66		66		71.5		67	
Sex								
Female	15	25.9	5	17.9	2	50	22	24.4
Male	43	74.1	23	82.1	2	50	68	75.6
Allograft								
Kidney	42	72.4	22	78.6	3	75.0	67	74.4
Liver	10	17.2	4	14.3	0	0.0	14	15.6
Heart	5	8.6	1	3.6	1	25.0	7	7.8
Lung	0	0.0	1	3.6	0	0.0	1	1.1
Heart + Kidney	1	1.7	0	0.0	0	0.0	1	1.1
First-line ICI								
PD-1	33	56.9	27	96.4	3	75.0	63	70.0
Pembrolizumab	25	43.1	11	39.3	1	25.0	37	41.1
Nivolumab	8	13.8	5	17.9	2	50.0	15	16.7
Cemiplimab	0	0.0	11	39.3	0	0.0	11	12.2
CTLA-4								
Ipilimumab	21	36.2	0	0.0	0	0.0	21	23.3
PD-L1								
Avelumab	0	0.0	0	0.0	1	25.0	1	1.1
Multiple								
CTLA-4 + PD-1	4	6.9	1	3.6	0	0.0	5	5.6
Second-line ICI								
PD-1	9	15.5	0	0.0	0	0.0	9	10.0
CTLA-4	1	1.7	0	0.0	0	0.0	1	1.1
CTLA-4 + PD-1	2	3.4	1	3.6	1	25.0	4	4.4
# Immunosuppressants								
0	1	1.7	1	3.6	1	25.0	3	3.3
1	27	46.6	7	25.0	1	25.0	35	38.9
2	22	37.9	16	57.1	2	50.0	40	44.4
3	8	13.8	4	14.3	0	0.0	12	13.3

cSCC, cutaneous squamous cell carcinoma; MCC, Merkel cell carcinoma; ICI, immune checkpoint inhibitor.

### Type of ICI for entire cohort

Of the 90 patients, 33 (36.7%) received prior non-ICI systemic therapy, 37 (41.1%) were treatment-naive, and the remaining 20 cases did not specify. The first-line immunotherapy most commonly consisted of anti-PD-1 monotherapy (*n* = 63, 70.0%) including pembrolizumab (*n* = 37, 41.1%), nivolumab (*n* = 15, 16.7%), and cemiplimab (*n* = 11, 12.2%). One patient received avelumab (*n* = 1, 1.1%). Twenty-one patients initially received single agent ipilimumab (23.3%). Five patients were initially treated with a combination of ipilimumab and a PD-1 inhibitor. Additionally, 14 patients (15.5%) were treated with second-line immunotherapy with an alternative ICI regimen due to disease progression on the initial ICI regimen. Median time from transplantation to initiation of ICI was 9 years (ranging from 0.2 to 32 years) in the 70 cases that reported it.

### Response to ICI for entire cohort

Objective responses to any ICI regimen for the entire cohort were: 17.8% CR (*n* = 16), 23.3% PR (*n* = 21), 7.8% SD (*n* = 7), and 48.9% PD (*n* = 44) for an objective response rate (ORR) (CR + PR) of 41.1%. One patient had a mixed response and one patient was considered unevaluable due to the patient expiring from graft failure before disease response could be evaluated. The majority of patients were treated with PD-1 inhibitors as first line ICI as opposed to ipilimumab monotherapy or ipilimumab plus nivolumab, with ORRs of 46%, 28.6%, and 40.0%, respectively. There were no significant differences in ORR between patients treated first-line with PD-1inhibitor vs. ipilimumab, PD-1 inhibitor monotherapy vs. ipilimumab plus nivolumab and ipilimumab monotherapy vs. ipilimumab plus a PD-1 inhibitor (*p* = 0.20, *p* = 1 and *p* = 0.63, respectively). Of note, there were 9 patients who had disease progression on ipilimumab and were started on a second-line PD-1 inhibitor, with an ORR of 44.4% (4/9) on the second-line agent. Of the patients who received a first-line PD-1 inhibitor, 4 were treated with second-line combination ipilimumab and nivolumab (ORR 25%, 1/4) while 1 was treated with second-line ipilimumab (ORR 0%, 0/1).

By tumor type, ORRs were 64.3% (18/28) for cSCC, 31% (18/58) for melanoma, and 25.0% (1/4) for MCC (Table 2). By type of SOT, ORRs were 57.1% for liver, 41.8% for kidney, and 0% for heart. The difference in ORR between patients with kidney and liver transplants was not significant (*p* = 0.38). However, as there was no tumor response in patients with heart transplants, the ORR was significantly higher when compared to patients with kidney (*p* = 0.04) and liver (*p* = 0.02) transplants.

**Table 2:** Response rate for tumor types.

	Melanoma		cSCC		MCC		All	
	*n*	%	*n*	%	*n*	%	*n*	%
Total	58	64.4	28	31.1	4	4.4	90	
ORR	18	31.0	18	64.3	1	25.0	37	41.1
CR	5	8.6	10	35.7	1	25.0	16	17.8
PR	13	22.4	8	28.6	0	0.0	21	23.3
PD	33	56.9	8	28.6	3	75.0	44	48.9
SD	5	8.6	2	7.1	0	0.0	7	7.8
Mixed	1	1.7	0	0.0	0	0.0	1	1.1
Nonevaluable	1	1.7	0	0.0	0	0.0	1	1.1

### SOT recipients with melanoma

The most common type of SOT in patients with melanoma was kidney (*n* = 42, 72.4%), followed by liver (*n* = 10, 17.2%), heart (*n* = 5, 8.6%), then heart and kidney (*n* = 1, 1.7%). The first line ICI regimen for melanoma patients was either ipilimumab (*n* = 21, 36.2%), a PD-1 inhibitor (*n* = 33, 56.9%), or a combination of ipilimumab and anti-PD-1 (*n* = 4, 6.9%). There were 9 patients who had disease progression on ipilimumab and were then treated with a second-line PD-1 inhibitor. Tumor responses were CR (*n* = 5, 8.6%), PR (*n* = 13, 22.4%), SD (*n* = 5, 8.6%), PD (*n* = 33, 56.9%), or mixed response (*n* = 1, 1.7%) for an ORR of 31%. There were 24 cases (41.4%) of acute graft rejection and 14 of those cases (58.3%) resulted in graft failure while the other 10 allografts were rescued with temporary discontinuation of ICI and immunosuppression.

### SOT recipients with cSCC

Twenty-eight patients (31.1%) had cSCC and the vast majority had a renal transplant (*n* = 22, 78.6%) followed by liver (*n* = 4, 14.3%), heart (*n* = 1, 3.6%), and lung (*n* = 1, 3.6%) transplants. All patients received frontline PD-1 inhibition except for one patient who received ipilimumab and nivolumab which was administered after disease progression on carboplatin and taxol, and then cetuximab. Tumor responses were CR (*n* = 10, 35.7%), PR (*n* = 8, 28.6%), SD (*n* = 2, 7.1%), or PD (*n* = 8, 28.6%) for an ORR of 64.3%. There were 9 cases (32.1%) of acute graft rejection and 6 of those cases (66.7%) resulted in graft failure.

### SOT recipients with MCC

Three patients with MCC were treated with a PD-1 inhibitor and one received a PD-L1 inhibitor. Tumor responses were CR (*n* = 1, 25%) or PD (*n* = 3, 75%) with an ORR of 25%. There was 1 case of acute kidney rejection that eventually progressed to graft failure.

### Impact of ICI on graft in the entire cohort

There were 34 cases (37.8%) of acute graft rejection of which 21 resulted in graft failure (61.8%). In the overall cohort, 21/90 (23.3%) patients had graft failure. On average, graft rejection occurred 49 days after ICI initiation (ranging from 4 to 240 days), with a median of 28.5 days. Patients treated with a combination of CTLA-4 and PD-1 inhibitors (4/5) had the highest rate of graft rejection at 80%, followed by graft rejection rates of 36.5% (23/63) for first-line PD-1 inhibitors, and 14.3% (3/21) for first-line ipilimumab (Table 3). In addition, of the 9 patients who were treated with ipilimumab then a PD-1 inhibitor for second-line treatment, 4 of them experienced graft rejection after the first dose of the PD-1 inhibitor while 1 experienced graft rejection after the 4th cycle of ipilimumab prior to starting the PD-1 inhibitor. There was 1 patient who was treated with a PD-1 inhibitor followed by second line ipilimumab and 3 patients who were treated with a PD-1 inhibitor followed by second line ipilimumab and nivolumab. None of those 4 patients experienced graft rejection. Graft rejection rates were higher with combination ipilimumab plus nivolumab compared to first-line ipilimumab monotherapy (*p* = 0.01). There was no difference in rates of rejection for patients treated with anti-PD-1 vs. ipilimumab (*p* = 0.06) or ipilimumab plus nivolumab vs. anti-PD-1 monotherapy (*p* = 0.08). The one patient treated with a PD-L1 inhibitor did not have a graft rejection. Rates of acute graft rejection and failure were 41.8% (*n* = 28) and 26.9% (*n* = 18) for kidney transplants, 28.6% (*n* = 4) and 21.4% (*n* = 3) for liver transplant, and 14.3% (*n* = 1) and 0% for heart transplant, respectively. There were no significant differences between graft rejection rates by transplant type (kidney vs. liver *p* = 0.55, kidney vs. heart *p* = 0.23, and liver vs. heart *p* = 0.62). Mean time from transplant to ICI initiation for patients with no acute graft rejection vs. acute rejection was 9.3 years and 11.7 years respectively.

**Table 3:** Rates of graft rejection and failure by ICI.

	Total		ORR		Graft rejection		Graft failure	
	*n*	%	*n*	%	*n*	%	*n*	%
First-line ICI	90		37	41.1	30	33.3	18	20.0
CTLA-4	21	23.3	6	28.6	3	14.3	1	4.8
PD-1	63	70.0	29	46.0	23	36.5	15	23.8
PD-L1	1	1.1	0	0.0	0	0.0	0	0.0
CTLA-4 + PD-1	5	5.6	2	40.0	4	80.0	2	40.0
Second-line ICI	14		5	35.7	4	28.6	3	21.4
CTLA-4 then PD-1^a^	9	64.3	4	44.4	4	44.4	3	33.3
PD-1 then CTLA-4^a^	1	7.1	0	0.0	0	0.0	0	0.0
PD-1 then CTLA-4 + PD-1^a^	4	28.6	1	25.0	0	0.0	0	0.0

^a^ ORR is based on tumor response to second-line ICI.

### Number and type of immunosuppressive agents for SOT

At the time of ICI initiation, patients were maintained on either 0 (*n* = 3), 1 (*n* = 35), 2 (*n* = 40), or 3 (*n* = 12) of the following immunosuppressive agents for their SOT: corticosteroids (prednisone), calcineurin inhibitors (tacrolimus, cyclosporine), mTOR inhibitors (sirolimus, everolimus), mycophenolic acid, and azathioprine.

The three patients who were not on any immunosuppressive agents at the time of ICI initiation all experienced graft rejection and two of those patients then experienced graft failure. Patients treated with 1, 2, and 3 immunosuppressive agent(s) had a graft rejection rate of 45.7% (16/35), 30% (12/40), and 25% (3/12), respectively and a graft failure rate of 31.4% (11/35), 17.5% (7/40), and 8.3% (1/12), respectively (Table 4). Patients on 2 or 3 immunosuppressive agents had lower rates of graft rejection compared to patients on 0 immunosuppressants (*p* = 0.04 and 0.04, respectively). There were no statistically significant differences in graft rejection rates when comparing patients treated with 1 vs. 2 (*p* = 0.23), 1 vs. 3 (*p* = 0.31), or 2 vs. 3 (*p* = 1.0) immunosuppressive agents, nor any differences in graft failure rates (*p* = 0.18, 0.15, and 0.66 respectively).

**Table 4:** Rates of graft rejection and failure by immunosuppressants.

	Total		ORR		Graft rejection		Graft failure	
	*n*	%	*n*	%	*n*	%	*n*	%
Total	90		37	42.2	34	37.8	21	23.3
No agent	3	3.3	2	66.7	3	100.0	2	66.7
Single agent	35	38.9	15	42.9	16	45.7	11	31.4
Corticosteroids	17	48.6	10	58.8	9	52.9	7	41.2
Calcineurin inhibitor	11	31.4	3	27.3	3	27.3	2	18.2
mTOR inhibitor	7	20.0	2	28.6	4	57.1	2	28.6
MPA	0	0.0	0	0.0	0	0.0	0	0.0
Aza	0	0.0	0	0.0	0	0.0	0	0.0
Dual agents	40	44.4	18	45.0	12	30.0	7	17.5
Calcineurin inhibitor + MPA	5	12.5	1	20.0	3	60.0	1	20.0
Calcineurin inhibitor + mTOR inhibitor	1	2.5	1	100.0	0	0.0	0	0.0
Calcineurin inhibitor + Corticosteroid	13	32.5	7	53.8	4	30.8	4	30.8
Corticosteroid + MPA	2	5.0	1	50.0	0	0.0	0	0.0
Corticosteroid + mTOR inhibitor	14	35.0	5	35.7	3	21.4	1	7.1
mTOR inhibitor + MPA	4	10.0	3	75.0	1	25.0	0	0.0
mTOR inhibitor + Aza	1	2.5	0	0.0	1	100.0	1	100.0
Triple agents	12	13.3	2	16.7	3	25.0	1	8.3
Corticosteroid + Calcineurin inhibitor + MPA	5	5.6	1	20.0	2	40.0	1	20.0
Corticosteroid + mTOR inhibitor + MPA	4	4.4	0	0.0	0	0.0	0	0.0
Corticosteroid + mTOR inhibitor + Aza	2	2.2	1	50.0	0	0.0	0	0.0
Corticosteroid + Calcineurin inhibitor + mTOR inhibitor	1	1.1	0	0.0	1	100.0	0	0.0

In patients treated with only a single immunosuppressive agent, those on prednisone monotherapy had the best response to ICI (ORR 58.8%), but also had a higher graft rejection rate of 52.9% and a graft failure rate of 41.2%. When comparing monotherapies, calcineurin inhibitors had the lowest rate of graft rejection (27.3%) and graft failure (18.2%). In patients receiving two or more immunosuppressive agents, those on an mTOR inhibitor and mycophenolic acid had the highest ORR at 75% (3/4), and the lowest rate of graft failure at 0%. Patients treated with both prednisone and an mTOR inhibitor also had lower rates of graft rejection (21.4%, 3/14) and failure (7.1%, 1/14) (Table 4).

In this series, it is difficult to evaluate whether a modification to the immunosuppressive regimen affected risk of graft rejection. Thirty-nine patients underwent a change in their immunosuppressive regimen prior to starting ICI therapy and 19 (48.7%) developed graft rejection. However, out of the 29 patients who had no change in their immunosuppressive regimen, 7 (24.1%) developed graft rejection in comparison. There was a statistically significant difference in rate of graft rejection between these two groups (*p* = 0.047).

Patients with melanoma were numerically more likely to have acute graft rejection than those with cSCC, while patients with cSCC were more likely to have their acute graft rejection progress to graft failure but the differences were not significant (*p* = 0.48 and *p* = 1). Liver transplant recipients tend to have lower rates of graft rejection and clinically, and it is possible that the selection of the type of and number of immunosuppressive agents may differ by type of transplant (71). In this series, patients with liver transplants received single agent immunosuppression (64.3%) at a numerically higher rate than patients with kidney transplants (31.3%), who were more commonly on 2 immunosuppressive agents at ICI initiation (50.7%). By type of SOT, rates of rejection were 41.8% (28/67), 28.6% (4/14), and 14.3% (1/7) for kidney, liver, and heart respectively and proceeded to graft failure in 64.3% (18/28), 75% (3/4), and 0% (0/1), respectively. Despite the difference in number of immunosuppressive agents, there were no significant differences in rate of graft rejection or graft failure when comparing liver to kidney transplants (*p* = 0.55 and *p* = 1, respectively).

The most common cause of death was disease progression, not graft failure, as has been commented on in another study (72). Of the 49 patients who died, 41 patients died due to progression of their underlying disease whereas 6/49 died from graft failure alone or a combination of graft failure and progression of disease. Of note, since the majority of patients received kidney transplants, if the allograft failed, patients underwent dialysis, which bias these results, and long term follow-up is not available. The two patients who declined dialysis passed away.

## Discussion

Use of ICI in SOT with cancer is a clinical challenge due to the risks of life-threatening graft rejection. This retrospective review represents one of the largest collections of SOT patients with advanced skin cancer treated with an ICI, mostly anti-PD-1 monotherapy. It also adds an additional 4 cases to the literature from our single institution experience. Tumor stabilization or regression was noted in 48.9% of cases and the ORR was 41.1% for the entire cohort. Responses to anti-PD-1 in SOT patients with cSCC (ORR 60.7%) were higher than that reported in clinical trials of non-SOT patients (ORR 35%–50%) and higher than in other case series of SOT patients with cSCC (73–75). Patient or tumor-based characteristics of individual cSCC patients included here that may have accounted for this discrepancy are not available. SOT patients with melanoma had similar response rates to anti-PD-1 compared with responses in non-SOT patients treated with nivolumab or pembrolizumab (76, 77). There were too few cases of SOT patients with MCC to draw conclusions regarding response rates. The durability of responses and long-term overall survival is unable to be assessed due to the retrospective nature of the study and because the data was available only through published case series, with the exception of 4 patients from our institution. However, this study confirms the efficacy of ICI for advanced skin cancers in the SOT population, which has been highlighted in several other series (48, 70).

Despite the relatively preserved activity of ICI in skin cancer patients with SOT, graft rejection occurred in 37.8% of patients and more than half of those (61.8%) progressed to graft failure and either became reliant on hemodialysis, in the setting of kidney transplant failure, or passed away (48). There were no differences in rejection rates by type of transplant, but 4 out of 5 (80%) patients who were treated with a combination of ipilimumab and nivolumab experienced acute rejection and 2 out of 5 (40%) had graft failure. However, there were three SOT patients who were first treated with a PD-1 inhibitor and then followed by a combination of ipilimumab and nivolumab who did not develop graft rejection. The sample size in this study is too small to draw conclusions regarding impact of dual checkpoint inhibition on graft function (59). Only one patient was treated with a PD-L1 inhibitor in our cohort which limits our assessment of whether rates of graft rejection differ between PD-1 and PD-L1 inhibitors. One retrospective study included 6 kidney transplant patients, who did not fit our inclusion criteria, treated with anti-PD-L1 and report a graft rejection rate of 0% (75). Graft rejection in our series occurred in a numerically higher proportion of patients who received anti-PD-1 monotherapy compared to ipilimumab monotherapy, but the difference was not statistically significant and there have been conflicting reports in other retrospective series (56, 75, 78). Prior series have similarly and consistently shown that graft rejection for SOT patients occurs in about 40% of cases, however rates of graft failure are more variable (48, 56, 70, 72, 79). Despite high rates of graft rejection, cause of death was most commonly from disease progression. This may be impacted by the ability of patients with renal transplant rejection to be rescued with dialysis (70, 72, 80). Finally, there was no significant difference in time from transplant and initiation of ICI in patients who had graft rejection and those who did not. While some studies show higher rejection rates if ICI is administered earlier after a transplant, other studies have not verified that correlation (21, 75, 81).

The optimal approach to immunosuppressive regimens in SOT receiving ICI is unknown but being studied in several clinical trials. Although our study found that rates of graft rejection were higher in patients who had modifications to their immunosuppressive regimen prior to starting ICI therapy compared to those who did not have a change in their immunosuppressive regimen, not all cases reported on whether changes were made. Additionally, there are multiple confounding factors that need to be accounted for to better evaluate this question. Interestingly, in a phase I clinical trial studying pembrolizumab in kidney transplant recipients, it was also noted that maintaining baseline immunosuppression before ICI may reduce the risk of graft rejection (82). However, this requires further study. Tailoring the immunosuppression for SOT patients receiving ICI should be carefully discussed between the oncology and transplant teams to modify risks and clinical trial participation, where able, is encouraged. In our series, patients receiving no immunosuppression had the best tumor responses whereas patients on three immunosuppressive agents were poorly responsive to ICI, consistent with other studies (83, 84). Rates of graft failure were clearly inversely correlated with number of immunosuppressive agents, ranging from only 8.3% with 3 agents to 31.4% with one agent.

Type and/or combination of immunosuppressive agent(s) likely also matters. In patients treated with a single immunosuppressive agent, calcineurin inhibitors were associated with the lowest proportion of graft rejection (27.3%) and graft failure (18.2%), as previously reported, however tumor responses were also lower (ORR 27.3%) (70, 84). A clinical trial (NCT03816332) studied the efficacy of an ipilimumab, nivolumab, prednisone and tacrolimus regimen in patients with kidney transplants and advanced skin cancers, although in the small sample size, tacrolimus and prednisone did not prevent rejection in 2/8 patients (85). In our series, while tumor responses were highest in patients receiving prednisone only (ORR 58.8%), rates of graft rejection (52.9%) and failure (41.2%) were much higher. In patients receiving dual agent immunosuppression, the regimen that suggested the best balanced tumor response with lower risk of graft rejection or failure was prednisone plus an mTOR inhibitor. This finding is supported by results of CONTRAC-1 (NCT04339062), a clinical trial in which SOT patients with cSCC were treated with cemiplimab. The immunosuppressive regimen was standardized and involved administration of mTOR inhibition with a prednisone taper surrounding each cycle of cemiplimab (40 mg on days 1–3, 20 mg on days 4–6, and 10 mg on days 7–20). Preliminary results from the study showed ORR 50% in 8 evaluable patients and no kidney allograft rejection or loss (86).

The potential benefit of immunosuppressive regimens that contain mTOR inhibitors has been explored in another multi-center retrospective cohort study in which mTOR inhibitors were associated with lower rates of kidney graft rejection for SOT recipients treated with ICIs (75). In addition, mTOR inhibitors may have potential anti-tumor properties for post-transplant patients with cSCC, which may partially explain the improved ORR for patients with cSCC compared to melanoma in our study (87). Two case reports have further delved into the role of mTOR inhibitors in uncoupling the efficacy and toxicity of PD-1 inhibition, but the results appear inconclusive due to the limited sample size. Although one study notes that mTOR inhibitors may abate pembrolizumab associated T-cell activation, another notes that alloreactive T-cells may still be present despite persistent mTOR immunosuppression and may be induced by PD-1 inhibitors (49, 88). Future studies should focus on strategies to optimize immunosuppression for the allograft so that T-cell responses are directed towards tumor rather than both tumor and allograft.

This study has multiple limitations. This was a retrospective review of published cases from the literature in addition to cases reported from our own institution. The effect of publication bias here cannot be understated and may underestimate the incidence of graft rejection on ICI initiation. Cases in the literature that documented graft rejection but did not report on ICI responses were excluded. Differences in survival cannot be determined as the follow-up for each study was variable or not reported. Although we adhered to standard inclusion criteria for selection of published articles studied here, the actual treatment approaches and diagnosis and management of graft rejection or failure were individualized. Due to the overall small sample size, although Fisher's exact test was used, statistical analysis was largely descriptive and multivariable analysis was not performed. Prospective controlled studies are needed to confirm any trends observed here.

Overall, the use of ICI in SOT patients with skin cancer requires thorough multidisciplinary discussion, consideration of other available treatment options, if effective, and possible modification of the patient's immunosuppressive regimen to decrease risk of graft rejection and/or failure, however this requires further study. Risks and benefits including the possibility of fatal graft failure must be discussed at length with each patient. ICI remains the standard of care for advanced skin cancers and has the highest potential for long-term durable responses. In many cases, risks of administration of ICI in SOT patients may be necessary when balancing the risks of death from progressive skin cancer. Clinical trials are ongoing to better understand how to optimize immunomodulation in this population. The number of immunosuppressants and the biology of each type of organ transplant could affect rates of rejection and further research must be done to understand the impact of ICIs in an organ-specific manner as well. Strategies to prevent and manage graft rejection should also be studied, for example, with use of IL-6 antibodies or other agents. Donor-derived cell-free DNA (dd-cfDNA) may be a useful biomarker in cancer patients treated with ICI to detect acute rejection (50, 64). Although this was a population typically excluded from clinical trials, as the numbers of SOT patients increase, more research is needed to address this unique population.

## Data Availability

The original contributions presented in the study are included in the article/**Supplementary Material**, further inquiries can be directed to the corresponding author.

## References

[1] https://unos.org/news/2022-organ-transplants-again-set-annual-records/.

[2] Neergaard L. US counts millionth organ transplant while pushing for more. New York: Associated Press News. (2022). Available at: https://apnews.com/article/science-health-organ-transplants-government-and-politics-308bfae0c70c3377d595b9a0a3a5a381

[3] Penn I. Post-transplant malignancy: the role of immunosuppression. Drug Saf. (2000) 23(2):101–13. 10.2165/00002018-200023020-00002 10945373

[4] Grulich AE van Leeuwen MT Falster MO Vajdic CM. Incidence of cancers in people with HIV/AIDS compared with immunosuppressed transplant recipients: a meta-analysis. Lancet. (2007) 370(9581):59–67. 10.1016/S0140-6736(07)61050-2 17617273

[5] Engels EA Pfeiffer RM Fraumeni JF Jr. Kasiske BL Israni AK Snyder JJ Spectrum of cancer risk among US solid organ transplant recipients. JAMA. (2011) 306(17):1891–901. 10.1001/jama.2011.1592 22045767 PMC3310893

[6] Buell JF Gross TG Woodle ES. Malignancy after transplantation. Transplantation. (2005) 80(2 Suppl):S254–64. 10.1097/01.tp.0000186382.81130.ba 16251858

[7] O’Reilly Zwald F Brown M. Skin cancer in solid organ transplant recipients: advances in therapy and management: part I. Epidemiology of skin cancer in solid organ transplant recipients. J Am Acad Dermatol. (2011) 65(2):253–61. 10.1016/j.jaad.2010.11.062 21763561

[8] Greenberg JN Zwald FO. Management of skin cancer in solid-organ transplant recipients: a multidisciplinary approach. Dermatol Clin. (2011) 29(2):231–41, ix. 10.1016/j.det.2011.02.004 21421148

[9] Euvrard S Kanitakis J Claudy A. Skin cancers after organ transplantation. N Engl J Med. (2003) 348(17):1681–91. 10.1056/NEJMra022137 12711744

[10] Garrett GL Blanc PD Boscardin J Lloyd AA Ahmed RL Anthony T Incidence of and risk factors for skin cancer in organ transplant recipients in the United States. JAMA Dermatol. (2017) 153(3):296–303. 10.1001/jamadermatol.2016.4920 28097368

[11] Vaddepally RK Kharel P Pandey R Garje R Chandra AB. Review of indications of FDA-approved immune checkpoint inhibitors per NCCN guidelines with the level of evidence. Cancers (Basel). (2020) 12(3):738. 10.3390/cancers12030738 32245016 PMC7140028

[12] Hwu P. Treating cancer by targeting the immune system. N Engl J Med. (2010) 363(8):779–81. 10.1056/NEJMe1006416 20818880

[13] Li W Zheng XX Kuhr CS Perkins JD. CTLA4 engagement is required for induction of murine liver transplant spontaneous tolerance. Am J Transplant. (2005) 5(5):978–86. 10.1111/j.1600-6143.2005.00823.x 15816877

[14] Wolchok JD Chiarion-Sileni V Gonzalez R Grob JJ Rutkowski P Lao CD Long-term outcomes with nivolumab plus ipilimumab or nivolumab alone versus ipilimumab in patients with advanced melanoma. J Clin Oncol. (2022) 40(2):127–37. 10.1200/JCO.21.02229 34818112 PMC8718224

[15] Grob JJ Gonzalez R Basset-Seguin N Vornicova O Schachter J Joshi A Pembrolizumab monotherapy for recurrent or metastatic cutaneous squamous cell carcinoma: a single-arm phase II trial (KEYNOTE-629). J Clin Oncol. (2020) 38(25):2916–25. 10.1200/JCO.19.03054 32673170 PMC7460151

[16] Nghiem PT Bhatia S Lipson EJ Kudchadkar RR Miller NJ Annamalai L PD-1 blockade with pembrolizumab in advanced Merkel-cell carcinoma. N Engl J Med. (2016) 374(26):2542–52. 10.1056/NEJMoa1603702 27093365 PMC4927341

[17] Murakami N Riella LV. Co-inhibitory pathways and their importance in immune regulation. Transplantation. (2014) 98(1):3–14. 10.1097/TP.0000000000000169 24978034

[18] Riella LV Watanabe T Sage PT Yang J Yeung M Azzi J Essential role of PDL1 expression on nonhematopoietic donor cells in acquired tolerance to vascularized cardiac allografts. Am J Transplant. (2011) 11(4):832–40. 10.1111/j.1600-6143.2011.03451.x 21401869

[19] Yang J Popoola J Khandwala S Vadivel N Vanguri V Yuan X Critical role of donor tissue expression of programmed death ligand-1 in regulating cardiac allograft rejection and vasculopathy. Circulation. (2008) 117(5):660–9. 10.1161/CIRCULATIONAHA.107.741025 18212277

[20] Smedman TM Line PD Guren TK Dueland S. Graft rejection after immune checkpoint inhibitor therapy in solid organ transplant recipients. Acta Oncol. (2018) 57(10):1414–8. 10.1080/0284186X.2018.1479069 29912605

[21] Johnson DB Sullivan RJ Menzies AM. Immune checkpoint inhibitors in challenging populations. Cancer. (2017) 123(11):1904–11. 10.1002/cncr.30642 28241095 PMC5445005

[22] Lipson EJ Bodell MA Kraus ES Sharfman WH. Successful administration of ipilimumab to two kidney transplantation patients with metastatic melanoma. J Clin Oncol. (2014) 32(19):e69–71. 10.1200/JCO.2013.49.2314 24493726 PMC4870592

[23] Morales RE Shoushtari AN Walsh MM Grewal P Lipson EJ Carvajal RD. Safety and efficacy of ipilimumab to treat advanced melanoma in the setting of liver transplantation. J Immunother Cancer. (2015) 3:22. 10.1186/s40425-015-0066-0 26082835 PMC4469313

[24] Qin R Salama AK. Report of ipilimumab in a heart transplant patient with metastatic melanoma on tacrolimus. Melanoma Manag. (2015) 2(4):311–4. 10.2217/mmt.15.27 30190859 PMC6096438

[25] Ranganath HA Panella TJ. Administration of ipilimumab to a liver transplant recipient with unresectable metastatic melanoma. J Immunother. (2015) 38(5):211. 10.1097/CJI.0000000000000077 25962109

[26] Alhamad T Venkatachalam K Linette GP Brennan DC. Checkpoint inhibitors in kidney transplant recipients and the potential risk of rejection. Am J Transplant. (2016) 16(4):1332–3. 10.1111/ajt.13711 26752406

[27] Gastman BR Ernstoff MS. Tolerability of immune checkpoint inhibition cancer therapy in a cardiac transplant patient. Ann Oncol. (2016) 27(12):2304–5. 10.1093/annonc/mdw293 27502714

[28] Herz S Hofer T Papapanagiotou M Leyh JC Meyenburg S Schadendorf D Checkpoint inhibitors in chronic kidney failure and an organ transplant recipient. Eur J Cancer. (2016) 67:66–72. 10.1016/j.ejca.2016.07.026 27614165

[29] Jose A Yiannoullou P Bhutani S Denley H Morton M Picton M Renal allograft failure after ipilimumab therapy for metastatic melanoma: a case report and review of the literature. Transplant Proc. (2016) 48(9):3137–41. 10.1016/j.transproceed.2016.07.019 27932166

[30] Lipson EJ Bagnasco SM Moore J Jr. Jang S Patel MJ Zachary AA Tumor regression and allograft rejection after administration of anti-PD-1. N Engl J Med. (2016) 374(9):896–8. 10.1056/NEJMc1509268 26962927 PMC4850555

[31] Ong M Ibrahim AM Bourassa-Blanchette S Canil C Fairhead T Knoll G. Antitumor activity of nivolumab on hemodialysis after renal allograft rejection. J Immunother Cancer. (2016) 4:64. 10.1186/s40425-016-0171-8 27777773 PMC5067882

[32] Spain L Higgins R Gopalakrishnan K Turajlic S Gore M Larkin J. Acute renal allograft rejection after immune checkpoint inhibitor therapy for metastatic melanoma. Ann Oncol. (2016) 27(6):1135–7. 10.1093/annonc/mdw130 26951628

[33] Deltombe C Garandeau C Renaudin K Hourmant M. Severe allograft rejection and autoimmune hemolytic Anemia after anti-PD1 therapy in a kidney transplanted patient. Transplantation. (2017) 101(9):e291. 10.1097/TP.0000000000001861 29633980

[34] Dueland S Guren TK Boberg KM Reims HM Grzyb K Aamdal S Acute liver graft rejection after ipilimumab therapy. Ann Oncol. (2017) 28(10):2619–20. 10.1093/annonc/mdx281 28961840

[35] Kittai AS Oldham H Cetnar J Taylor M. Immune checkpoint inhibitors in organ transplant patients. J Immunother. (2017) 40(7):277–81. 10.1097/CJI.0000000000000180 28719552

[36] Kwatra V Karanth NV Priyadarshana K Charakidis M. Pembrolizumab for metastatic melanoma in a renal allograft recipient with subsequent graft rejection and treatment response failure: a case report. J Med Case Rep. (2017) 11(1):73. 10.1186/s13256-017-1229-z 28315636 PMC5357565

[37] Miller DM Trowbridge RM Desai A Drews RE. Kaposi’s varicelliform eruption in a patient with metastatic melanoma and primary cutaneous anaplastic large cell lymphoma treated with talimogene laherparepvec and nivolumab. J Immunother Cancer. (2018) 6(1):122. 10.1186/s40425-018-0437-4 30454071 PMC6245809

[38] Owonikoko TK Kumar M Yang S Kamphorst AO Pillai RN Akondy R Cardiac allograft rejection as a complication of PD-1 checkpoint blockade for cancer immunotherapy: a case report. Cancer Immunol Immunother. (2017) 66(1):45–50. 10.1007/s00262-016-1918-2 27771741 PMC5588660

[39] Schvartsman G Perez K Sood G Katkhuda R Tawbi H. Immune checkpoint inhibitor therapy in a liver transplant recipient with melanoma. Ann Intern Med. (2017) 167(5):361–2. 10.7326/L17-0187 28761949

[40] Winkler JK Gutzmer R Bender C Lang N Zeier M Enk AH Safe administration of an anti-PD-1 antibody to kidney-transplant patients: 2 clinical cases and review of the literature. J Immunother. (2017) 40(9):341–4. 10.1097/CJI.0000000000000188 29028789

[41] DeLeon TT Salomao MA Aqel BA Sonbol MB Yokoda RT Ali AH Pilot evaluation of PD-1 inhibition in metastatic cancer patients with a history of liver transplantation: the mayo clinic experience. J Gastrointest Oncol. (2018) 9(6):1054–62. 10.21037/jgo.2018.07.05 30603124 PMC6286929

[42] Grant MJ DeVito N Salama AKS. Checkpoint inhibitor use in two heart transplant patients with metastatic melanoma and review of high-risk populations. Melanoma Manag. (2018) 5(4):MMT10. 10.2217/mmt-2018-0004 30459942 PMC6240846

[43] Kuo JC Lilly LB Hogg D. Immune checkpoint inhibitor therapy in a liver transplant recipient with a rare subtype of melanoma: a case report and literature review. Melanoma Res. (2018) 28(1):61–4. 10.1097/CMR.0000000000000410 29140833

[44] Lesouhaitier M Dudreuilh C Tamain M Kanaan N Bailly E Legoupil D Checkpoint blockade after kidney transplantation. Eur J Cancer. (2018) 96:111–4. 10.1016/j.ejca.2018.03.019 29705511

[45] Sadaat M Jang S. Complete tumor response to pembrolizumab and allograft preservation in renal allograft recipient on immunosuppressive therapy. J Oncol Pract. (2018) 14(3):198–9. 10.1200/JOP.2017.027326 29257718

[46] Tio M Rai R Ezeoke OM McQuade JL Zimmer L Khoo C Anti-PD-1/PD-L1 immunotherapy in patients with solid organ transplant, HIV or hepatitis B/C infection. Eur J Cancer. (2018) 104:137–44. 10.1016/j.ejca.2018.09.017 30347289 PMC10176037

[47] Zehou O Leibler C Arnault JP Sayegh J Montaudie H Remy P Ipilimumab for the treatment of advanced melanoma in six kidney transplant patients. Am J Transplant. (2018) 18(12):3065–71. 10.1111/ajt.15071 30107088

[48] Abdel-Wahab N Safa H Abudayyeh A Johnson DH Trinh VA Zobniw CM Checkpoint inhibitor therapy for cancer in solid organ transplantation recipients: an institutional experience and a systematic review of the literature. J Immunother Cancer. (2019) 7(1):106. 10.1186/s40425-019-0585-1 30992053 PMC6469201

[49] Esfahani K Al-Aubodah TA Thebault P Lapointe R Hudson M Johnson NA Targeting the mTOR pathway uncouples the efficacy and toxicity of PD-1 blockade in renal transplantation. Nat Commun. (2019) 10(1):4712. 10.1038/s41467-019-12628-1 31624262 PMC6797722

[50] Hurkmans DP Verhoeven J de Leur K Boer K Joosse A Baan CC Donor-derived cell-free DNA detects kidney transplant rejection during nivolumab treatment. J Immunother Cancer. (2019) 7(1):182. 10.1186/s40425-019-0653-6 31300068 PMC6626432

[51] Lee BT Horwich BH Chopra S Ahearn A Han HH. Checkpoint inhibitor-induced rejection of a liver allograft: a combination of acute T cell-mediated and antibody-mediated rejection. Liver Transpl. (2019) 25(12):1845–8. 10.1002/lt.25622 31408574

[52] Singh P Visger Von J Prosek J Rovin B Pesavento TE Olencki T Preserved renal allograft function and successful treatment of metastatic merkel cell cancer post nivolumab therapy. Transplantation. (2019) 103(2):e52–3. 10.1097/TP.0000000000002502 30365468

[53] Bystrup Boyles T Schødt M Hendel HW Krarup-Hansen A Junker N. Pembrolizumab as first line treatment of merkel cell carcinoma patients–a case series of patients with various co-morbidities. Acta Oncol (Madr). (2020) 59(7):793–6. 10.1080/0284186X.2020.1747637 32285728

[54] Danesh MJ Mulvaney PM Murakami N Riella LV Silk AW Hanna GJ Impact of corticosteroids on allograft protection in renal transplant patients receiving anti-PD-1 immunotherapy. Cancer Immunol Immunother. (2020) 69(9):1937–41. 10.1007/s00262-020-02644-2 32588077 PMC7479641

[55] Hanna DL Law SJ Merrick SA Heptinstall L Bass P Dupont P The successful use of pembrolizumab in a renal transplant recipient with metastatic melanoma. Melanoma Res. (2020) 30(3):321–4. 10.1097/CMR.0000000000000651 31764435

[56] Kumar V Shinagare AB Rennke HG Ghai S Lorch JH Ott PA The safety and efficacy of checkpoint inhibitors in transplant recipients: a case series and systematic review of literature. Oncologist. (2020) 25(6):505–14. 10.1634/theoncologist.2019-0659 32043699 PMC7288631

[57] Owoyemi I Vaughan LE Costello CM Thongprayoon C Markovic SN Herrmann J Clinical outcomes of solid organ transplant recipients with metastatic cancers who are treated with immune checkpoint inhibitors: a single-center analysis. Cancer. (2020) 126(21):4780–7. 10.1002/cncr.33134 32786022 PMC8772343

[58] Soellradl I Kehrer H Cejka D. Use of ipilimumab and pembrolizumab in metastatic melanoma in a combined heart and kidney transplant recipient: a case report. Transplant Proc. (2020) 52(2):657–9. 10.1016/j.transproceed.2019.09.014 32044081

[59] Trager MH Coley SM Dube G Khan S Ingham M Samie FH Combination checkpoint blockade for metastatic cutaneous malignancies in kidney transplant recipients. J Immunother Cancer. (2020) 8(1):e000908. 10.1136/jitc-2020-000908 32503950 PMC7279669

[60] Venkatachalam K Malone AF Heady B Santos RD Alhamad T. Poor outcomes with the use of checkpoint inhibitors in kidney transplant recipients. Transplantation. (2020) 104(5):1041–7. 10.1097/TP.0000000000002914 31415036

[61] Brumfiel CM Patel MH Aqel B Lehrer M Patel SH Seetharam M. Immune checkpoint inhibitor therapy in a liver transplant recipient with autoimmune disease and metastatic cutaneous squamous cell carcinoma. JAAD Case Rep. (2021) 14:78–81. 10.1016/j.jdcr.2021.05.012 34277916 PMC8263514

[62] Gambichler T Hessam S Luttringhaus T Boms S. Prompt response of advanced cutaneous squamous cell carcinoma to cemiplimab in a kidney transplant patient receiving treatment with everolimus. Clin Exp Dermatol. (2021) 47(3):608–9. 10.1111/ced.15018 34767651

[63] Goodman AE Karapetyan L Pugliano-Mauro M Levenson JE Luke JJ. Case report: single dose anti-PD1 in a patient with metastatic melanoma and cardiac allograft. Front Immunol. (2021) 12:660795. 10.3389/fimmu.2021.660795 33828564 PMC8019780

[64] Lakhani L Alasfar S Bhalla A Aala A Rosenberg A Ostrander D Utility of serial donor-derived cell-free DNA measurements for detecting allograft rejection in a kidney transplant recipient after PD-1 checkpoint inhibitor administration. Transplant Direct. (2021) 7(2):e656. 10.1097/TXD.0000000000001113 33490381 PMC7817285

[65] Paoluzzi L Ow TJ. Safe administration of cemiplimab to a kidney transplant patient with locally advanced squamous cell carcinoma of the scalp. Curr Oncol. (2021) 28(1):574–80. 10.3390/curroncol28010057 33477979 PMC7903284

[66] Tan B Baxter M Casasola R. Acute renal transplant rejection following nivolumab therapy for metastatic melanoma. BMJ Case Rep. (2021) 14(2):e238037. 10.1136/bcr-2020-238037 33558380 PMC7872919

[67] Tsung I Worden FP Fontana RJ. A pilot study of checkpoint inhibitors in solid organ transplant recipients with metastatic cutaneous squamous cell carcinoma. Oncologist. (2021) 26(2):133–8. 10.1002/onco.13539 32969143 PMC7873324

[68] Bastos S Masmoudi W Pinard C Duval-Modeste AB Joly P Hebert V. Efficacy of nivolumab in the treatment of metastatic cutaneous squamous cell carcinoma in a kidney-transplant patient with a history of allograft rejection. Ann Dermatol Venereol. (2022) 149(3):198–9. 10.1016/j.annder.2022.01.003 35181155

[69] O'Connell KA Schmults CD. Treatment of metastatic cutaneous squamous cell carcinoma in a solid organ transplant recipient with programmed death-1 checkpoint inhibitor therapy. J Eur Acad Dermatol Venereol. (2022) 36(Suppl 1):45–8. 10.1111/jdv.17407 34855241

[70] Portuguese AJ Tykodi SS Blosser CD Gooley TA Thompson JA Hall ET. Immune checkpoint inhibitor use in solid organ transplant recipients: a systematic review. J Natl Compr Canc Netw. (2022) 20(4):406–416.e11. 10.6004/jnccn.2022.7009 35390767

[71] Abrol N Jadlowiec CC Taner T. Revisiting the liver’s role in transplant alloimmunity. World J Gastroenterol. (2019) 25(25):3123–35. 10.3748/wjg.v25.i25.3123 31333306 PMC6626728

[72] Fisher J Zeitouni N Fan W Samie FH. Immune checkpoint inhibitor therapy in solid organ transplant recipients: a patient-centered systematic review. J Am Acad Dermatol. (2020) 82(6):1490–500. 10.1016/j.jaad.2019.07.005 31302190

[73] Migden MR Rischin D Schmults CD Guminski A Hauschild A Lewis KD PD-1 blockade with cemiplimab in advanced cutaneous squamous-cell carcinoma. N Engl J Med. (2018) 379(4):341–51. 10.1056/NEJMoa1805131 29863979

[74] Hughes BGM Munoz-Couselo E Mortier L Bratland Å Gutzmer R Roshdy O Pembrolizumab for locally advanced and recurrent/metastatic cutaneous squamous cell carcinoma (KEYNOTE-629 study): an open-label, nonrandomized, multicenter, phase II trial. Ann Oncol. (2021) 32(10):1276–85. 10.1016/j.annonc.2021.07.008 34293460

[75] Murakami N Mulvaney P Danesh M Abudayyeh A Diab A Abdel-Wahab N A multi-center study on safety and efficacy of immune checkpoint inhibitors in cancer patients with kidney transplant. Kidney Int. (2021) 100(1):196–205. 10.1016/j.kint.2020.12.015 33359528 PMC8222056

[76] Robert C Long GV Brady B Dutriaux C Maio M Mortier L Nivolumab in previously untreated melanoma without BRAF mutation. N Engl J Med. (2015) 372(4):320–30. 10.1056/NEJMoa1412082 25399552

[77] Robert C Schachter J Long GV Arance A Grob JJ Mortier L Pembrolizumab versus ipilimumab in advanced melanoma. N Engl J Med. (2015) 372(26):2521–32. 10.1056/NEJMoa1503093 25891173

[78] Aguirre LE Guzman ME Lopes G Hurley J. Immune checkpoint inhibitors and the risk of allograft rejection: a comprehensive analysis on an emerging issue. Oncologist. (2019) 24(3):394–401. 10.1634/theoncologist.2018-0195 30413665 PMC6519766

[79] Rossi E Schinzari G Maiorano BA Esposito I Acampora A Romagnoli J Immune-checkpoint inhibitors in renal transplanted patients affected by melanoma: a systematic review. Immunotherapy. (2022) 14(1):65–75. 10.2217/imt-2021-0195 34751039

[80] Ferrándiz-Pulido C Leiter U Harwood C Proby CM Guthoff M Scheel CH Immune checkpoint inhibitors in solid organ transplant recipients with advanced skin cancers-emerging strategies for clinical management. Transplantation. (2023) 107(7):1452–62. 10.1097/tp.0000000000004459 36706163

[81] Au KP Chok KSH. Immunotherapy after liver transplantation: where are we now? World J Gastrointest Surg. (2021) 13(10):1267–78. 10.4240/wjgs.v13.i10.1267 34754394 PMC8554723

[82] Carroll RP Boyer M Gebski V Hockley B Johnston JK Kireta S Immune checkpoint inhibitors in kidney transplant recipients: a multicentre, single-arm, phase 1 study. Lancet Oncol. (2022) 23(8):1078–86. 10.1016/S1470-2045(22)00368-0 35809595

[83] d’Izarny-Gargas T Durrbach A Zaidan M. Efficacy and tolerance of immune checkpoint inhibitors in transplant patients with cancer: a systematic review. Am J Transplant. (2020) 20(9):2457–65. 10.1111/ajt.15811 32027461

[84] Alzahrani N Al Jurdi A Riella LV. Immune checkpoint inhibitors in kidney transplantation. Curr Opin Organ Transplant. (2023) 28(1):46–54. 10.1097/MOT.0000000000001036 36579684 PMC9811500

[85] Schenk KM Stein JE Chandra S Davar D Eroglu Z Khushalani NI Nivolumab (NIVO) + tacrolimus (TACRO) + prednisone (PRED) +/− ipilimumab (IPI) for kidney transplant recipients (KTR) with advanced cutaneous cancers. J Clin Oncol. (2022) 40(16_suppl):9507. 10.1200/JCO.2022.40.16_suppl.9507 PMC11677297 38252910

[86] Hanna GJ Dharaneeswaran HJ Giobbie-Hurder A Harran JJ Liao Z Pai L Cemiplimab for kidney organ transplant recipients with advanced cutaneous squamous cell carcinoma: CONTRAC-1. J Clin Oncol. (2023) 41(16_suppl):9519. 10.1200/JCO.2023.41.16_suppl.9519 PMC10950183 38252908

[87] Euvrard S Morelon E Rostaing L Goffin E Brocard A Tromme I Sirolimus and secondary skin-cancer prevention in kidney transplantation. N Engl J Med. (2012) 367(4):329–39. 10.1056/NEJMoa1204166 22830463

[88] Dunlap GS DiToro D Henderson J Shah SI Manos M Severgnini M Clonal dynamics of alloreactive T cells in kidney allograft rejection after anti-PD-1 therapy. Nat Commun. (2023) 14(1):1549. 10.1038/s41467-023-37230-4 36941274 PMC10027853

